# Tibetan Macaques with Higher Social Centrality and More Relatives Emit More Frequent Visual Communication in Collective Decision-Making

**DOI:** 10.3390/ani11030876

**Published:** 2021-03-19

**Authors:** Zifei Tang, Xi Wang, Mingyang Wu, Shiwang Chen, Jinhua Li

**Affiliations:** 1 School of Resources and Environmental Engineering, Anhui University, Hefei 230601, China; x18201004@stu.ahu.edu.cn; 2 International Collaborative Research Center for Huangshan Biodiversity and Tibetan Macaque Behavior Ecology, Hefei 230601, China; 15142344740@163.com (M.W.); chenwang1962@163.com (S.C.); 3 School of Life Sciences, Anhui University, Hefei 230601, China; 4 School of Life Sciences, Hefei Normal University, Hefei 230601, China

**Keywords:** Tibetan macaques (*Macaca thibetana*), collective decision-making, visual communication, social centrality, number of relatives

## Abstract

**Simple Summary:**

It is well known that visual communication plays an important role in collective decision-making. However, there is not much research on the influencing factors of visual signals, especially kinship and social relations. In this study, we not only confirmed the function of visual communication in collective decision-making, but also found the effect of kinship and social relations on visual communication. Tibetan macaques with higher social centrality and more relatives emit more frequent visual communication, providing a reference for further research on decision-making. Understanding the link between communication and decision-making can elucidate the powers of group maintenance in animal societies.

**Abstract:**

Animals on the move often communicate with each other through some specific postures. Previous studies have shown that social interaction plays a role in communication process. However, it is not clear whether the affinity of group members can affect visual communication. We studied a group of free-ranging Tibetan macaques (*Macaca thibetana*) at Huangshan Mountain, China, and answered whether and how social centrality or relatives matter in visual signals during group movement using Tobit regression modeling. All individuals emitted the signals of back-glances and pauses in collective movement. The emission of two signals decreased with the number of participants increased. The back-glance and pause signals emitted by the participating individuals were stronger as the position moved backward in the group. Sex, age, and rank had no significant influence on back-glance and pause signals. Individuals with higher social centrality would emit more pause signals, but social centrality had no effect on the back-glance signal. Individuals with more relatives in the group had more back-glance signals, but this had no effect on the pause signal. This study verifies that social centrality and the number of relatives have effects on visual signals in Tibetan macaques. We provide insights into the relationship between communication behaviors and group cooperation in social animals.

## 1. Introduction

To maintain group cohesion, social animals need to achieve collective movement in a cooperative manner, which facilitates information sharing, benefits reciprocity, and collective defense [1,2]. However, members often differ in age, sex, and needs, and then these differences generate divergent interests among individuals, which, in extreme cases, might lead to fragmentation and instability, and even deprive them of the benefits of group living [3,4]. During adaptive evolution, animals employ consensus decision-making to coordinate their actions on the move.

Consensus decision-making refers to the process by which members of a group achieve consistency in their activities through information exchange, where they decide when to leave and where to go [5]. To lead a successful collective movement, the initiator must use effective communication to evoke actions and behavioral imitations from other members and attract most of them to participate in the collective movement [6]. Meanwhile, other members of a collective movement can also communicate their preferences of movement direction and time to group mates, either through local communication among neighbors only [7] or through global communication in their group [8], ultimately achieving the consistency of members’ actions [2]. Clearly, the proper use of communication behaviors plays an important role in the success of consensus decision-making and the achievement of a collective movement [9].

Animals can signal departure to group members through visual signals, attracting group members to follow movements they initiate, negotiate the direction and timing of movements, and thus group movement [5]. For example, in group movements of bottlenose dolphins (*Tursiops truncatus*), the initiators recruit participants a lateral fin-swinging posture prior to group migration [10]. Visual communication in group movements has also been seen in studies of other animals. In domestic geese (*Anser domesticus*), a higher number of arousal behaviors (flutter wings on both sides) led to a larger number of individuals recruited [11]. European bisons (*Bison bonasus*) depart in a constant direction (without turning more than 45°) for at least 20 steps without stopping, with its head horizontal (without grazing) in order to recruit follower [12]; Spotted hyaenas (*Crocuta crocuta*) view bristled tails as initiation signal of collective movement [13].

Primates are highly cognitive and socially complex, with individuals possessing both local knowledge of the behavior of their neighbors and information about the environment holistically, thus making the functions of communicative behavior more diverse and complex [14]. For example, male barbary macaques (*Macaca sylvanus*) recruit neighboring individuals and monitor the activities of other group members when leading group movements [9]. At the same time, in primate collective movement, other members of the group may also influence the behavior of the initiator’s behavior. The negotiation process is not only signaling, but also a communicative response between members, that is, when initiators monitor the reluctance of group members to follow the movement, they increase the frequency of recruitment. As the number of participants gradually increases, the recruitment signals emitted by initiators decrease [14]. For example, in Tonkean macaques (*M. tonkeana*), the initiator glances back at other members of the group when leaving the group, pauses briefly while moving, and changes the speed of movement [15]. In addition, the involvement of individual relatives significantly reduces the release of initiator recruitment signals in red-tailed sportive lemurs (*Lepilemur ruficaudatus*) during travel to a new foraging site or habitat [16]. In recent years, research on communication behavior that occurs during primate consensus decision-making has become increasingly popular [17,18].

We previously conducted some collective decision-making research in Tibetan macaques (*M. thibetana*). For example, individuals with higher social centrality have a higher success rate of decision-making [19], and they will join group movement earlier. These individuals often decide whether to use selective mimetism or quorum [20]. We also found that female Tibetan macaques use their social networks to enhance the speed of collective decision-making, which may have associated fitness benefits [21], but there is no study on the relationship between communication behavior and behavioral decision-making. To clarify the factors that influence visual signals and how visual signals contribute to collective decision-making, we need to explore the potential link between communication and decision-making in Tibetan macaques.

We hypothesized that if back-glance and pauses were indeed used as communication signals (Hypothesis 1), then the signals would decrease significantly as the number of followers increases (Prediction 1).

Discrimination in factors, sex, age class, rank, number of relatives within the group, social centrality, and location in the movement queue were known to play an important role in signal transmission in primate groups. We hypothesized that these parameters would influence visual signal emissions (Hypothesis 2). Each factor above corresponds to one prediction, and there were altogether six predictions (Prediction 2–Prediction 7). That is to say, these six factors all have an impact on the release of visual signals.

## 2. Materials and Methods

### 2.1. Study Site and Subjects

We conducted this study from September 2019 to January 2020 in the Valley of the Wild Monkeys, Mt. Huangshan National Reserve in Anhui, China (30°29′ N, 118°10′ E) [22]. This location is a UNESCO World Heritage site, classified as such for biodiversity and cultural reasons, and is a popular tourist destination [23].

The research group was that of Tibetan macaques (Yulinkeng, YA1), a natural wild population. YA1 group has been carried out for more than 30 years since 1986 and it has kept records of the changes in the monkey troop throughout the year (such as birth, death, society, and migration) [24]. We can therefore identify all individuals in the troop according to their natural characteristics.

We provided for the monkeys with 3–4 kg of corn (artificially) at regular intervals (09:00, 11:00, 14:00, and 16:00) at a fixed, highly visible point [22,23], but their main food source was still natural wild plants. At the time of our study, the troop consisted of 35 individuals (10 adult males, 20 adult females, 3 sub-adult males, and 2 sub-adult females), and juvenile individuals and infant monkeys were not considered [19] (Supplementary material Table S1). Movement from the platform to the forest occurred several times a day, giving us the opportunity to observe collective decision-making during group movements [25].

### 2.2. Data Collection and Behavioral Definition

Group movements were observed and filmed continuously by three observers. We recorded each collective movement on a videotape (Canon EOS 550D; Canon Inc., Tokyo, Japan), and these videotapes were analyzed only by one person (Tang) [26]. Observations for the focal group lasted 6.5 h per day from 08:30 to 11:30 and from 14:00 to 17:30. The total focal sampling time of all individual was 180,000 s, with 10 rounds of focal for each individual. When some individuals fail to be recorded due to objective reasons, we finally use the weighted method to calculate. We recorded group movements and aggression-submission rounds using a digital voice recorder (SONY China, Beijing, China) using an all-occurrence sampling method [27]. The lack of movement in behavioral data was not considered in the analysis. For example, we discarded cases in which an individual had not been continuously observed, since we could not determine whether behaviors, such as pause or back-glance, might have been displayed [26]. We excluded movements caused by aggressively submissive interactions or sexual chases [28]. Focal animal sampling [27] was used to observe and collect data from 35 adult and sub-adult members in the monkey group, and the sampling time was 10 min each time. Detailed behavioral definitions are shown in Table 1 [10,19,24,25,28,29,30,31].

### 2.3. Data Analysis 

Based on the aggression-submission bouts and win-lose proportions, we calculated David’s Score, and then scored individuals’ dominance ranks by the DS, using the following steps: [32].

The proportion of wins by individual i in his interactions with another individual j (P_ij_) is the number of times that i defeats j (a_ij_) divided by the total number of interactions between i and j (n_ij_), that is,

P_ij_ = a_ij_/n_ij_. (1)

The proportion of losses by i in interactions with j,

P_ji_ = 1 − P_ij_. (2)

If n_ij_ = 0, then P_ij_ = 0 and P_ji_ = 0.

DS for each member, i, of a group is calculated with the following formula:

DS = W + W_2_ − l − l_2_ (3)

where W represents the sum of i’s P_ij_ values, W_2_ represents the summed W values (weighted by the appropriate P_ij_ values) of those individuals with whom i interacted, l represents the sum of i’s P_ji_ values, and l_2_ represents the summed l values (weighted by the appropriate P_ji_ values) of those individuals with whom i interacted [33,34].

To calculate the social centrality, we chose the eigenvector centrality as the index. The eigenvector centrality coefficient (ECC) is a parameter indicating the degree of connection of an individual in the group, calculated by the number and strength of the connections [35]. It also considers the identity of the partners it connects with [36]. Proximity is an important behavior of reflecting affiliative relations between group mates [24]. In this study, the dyadic association index (DAI) [27] based on proximity matrix was used to evaluate the affiliation among individuals in YA1 group. The calculation formula is as follows:

DAI_AB_ = ∑ (A + B) / ∑A + ∑B − ∑(A + B) (4)

where A and B represent the total time of focal animal sampling for individual A and individual B; A + B represents the total time of 1 m proximity for individual A and individual B.

We considered two individuals as related while belonging to the same matriline regardless of their degree of relatedness and addressed a coefficient equal to 1 to the dyad [26]. We corrected the coefficient of kinship as the number of individuals within a group that were linked by matrilineal kinship. In this study group, the number of relatives of the migrating individuals was recorded as 0.

We defined the departure latency, of every joiner, as the time elapsed between its departure and the time of the first departed individual. Using these latencies, we determined the order of individuals at each collective movement [26]. At departure, we attributed position 0 to the first departed individual and position j to the individual that joined the movement when j individuals were already participating. Thus,

j_max_ = N − 1 (5)

where N is the number of adult group members.

### 2.4. Statistical Analysis

Spearman’s correlation was used to test the relationship between the number of followers and the frequency of the initiator’s visual communication using SPSS (version 26.0) (SPSS Inc., Chicago, IL, USA). We calculated the eigenvector centrality coefficient of each individual using UCINET v6.2, and then drew the social network by NETDRAW v2.0. In this study, there were a lot of zero values for the back-glance and pause signals, and individuals were unnecessary to use visual signals in every single movement. To address the problem of too many zeros in the dependent variables, we used Tobit regression modeling [37]. The Tobit model was run in Stata v15.0 [37]. We used Cook’s distance to distinguish the outliers. After discrimination, we identified two outliers and eliminated them [38]. We then tested whether sex, age class, rank, social centrality, number of relatives, and position could affect the frequency of visual communication. The confidence interval of all tests was *p* < 0.05. ORIGIN v8.6 was used for drawing all figures.

### 2.5. Ethics Statement

This study complies with the regulations of the Chinese Wildlife Conservation Association regarding the ethical treatment of research subjects, and under the law of People’s Republic of China on the protection of wildlife. The study was fully observational, and our data collection did not affect the monkeys’ welfare. Huangshan Monkey Management Center and the Huangshan Garden Forest Bureau permitted us to conduct research at the field site.

## 3. Results

### 3.1. Distribution of Visual Signals during Collective Movements

During the study period, 126 successful collective movements involving 10 adult males, 20 adult females, 3 sub-adult males, and 2 sub-adult females were recorded. Among them, the number of collective movements with visual communication was 49, accounting for 38.9%, while the number of collective movements without visual communication was 77, accounting for 61.1%. The total number of visual signals was 521, including 246 back-glance signals (47.2%) and 275 pause signals (52.8%). All individuals were found to have back-glance and pausing behavior.

### 3.2. The Initiator’s Visual Signals Change with the Number of Followers

Pauses and back-glances were correlated for Tibetan macaques (Spearman’s rank correlation: *N* = 146, r = 0.25, *p* < 0.01). Indeed, it seems that the departed individual emitted back-glances when it paused during the observation period. However, even if these two variables are correlated, they are not collinear (collinearity diagnostics, VIF = 1.272; collinearity is a high correlation between two variables; the variance inflation factor (VIF) has to be >5 to consider two variables as collinear and thus dependent [39]). We can therefore consider the number of pauses and the number of back-glances as independent variables.

By counting 126 successful cluster campaigns, we recorded a total of 14 types of follower numbers, and some sequences were not recorded and therefore not analyzed. After classification, we calculated the average frequency of the initiator’s visual signal when the number of followers is different. (Supplementary material: Table S2).

**Figure 1:**
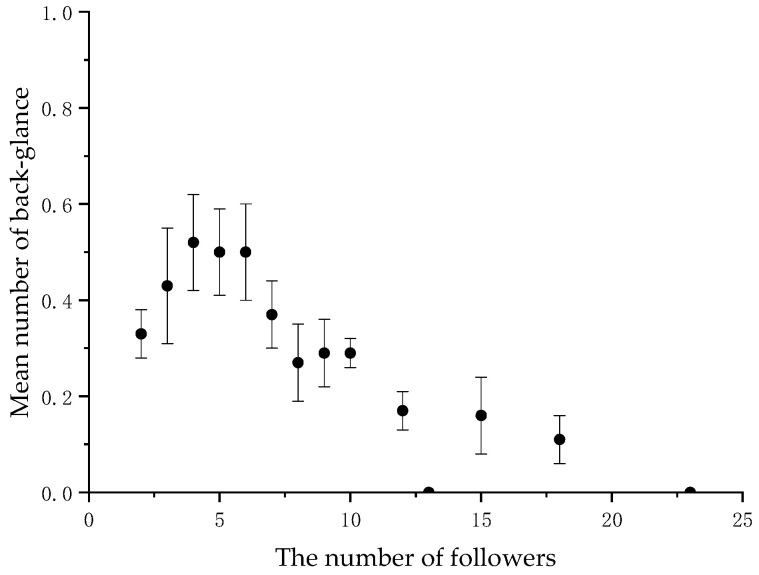
Mean frequency of the initiator’s back-glance per collective movement according to the number of followers.

**Figure 2:**
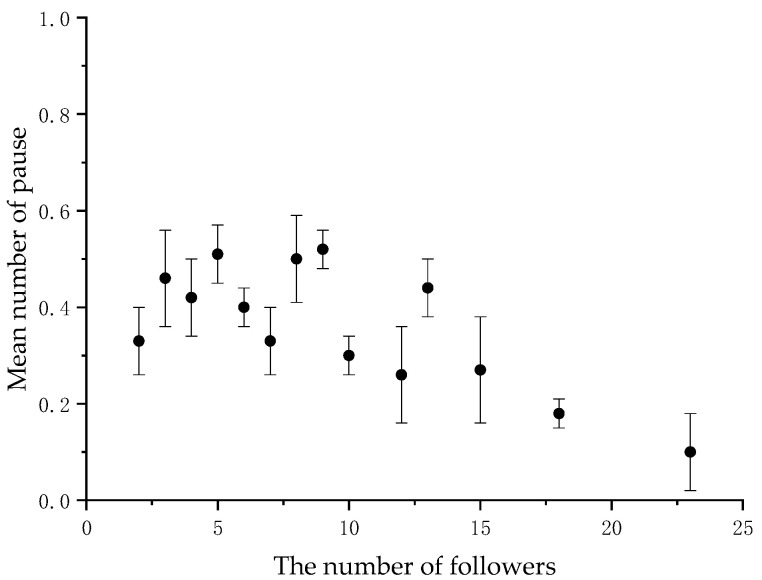
Mean frequency of the initiator’s pause per collective movement according to the number of followers.

When the number of followers varied, we calculated the average frequency of back-glances and pauses by the initiators at each level. There was a significant negative correlation between the initiator’s back-glances and the number of followers (*N* = 14, r = −0.873, *p* < 0.001) (Figure 1 and Table 2, and between the initiator’s pause and the number of followers (*N* = 14, r = −0.581, *p* < 0.05) (Figure 2 and Table 2 in the collective movements.

### 3.3. Factors Affecting Visual Signals

**Figure 3:**
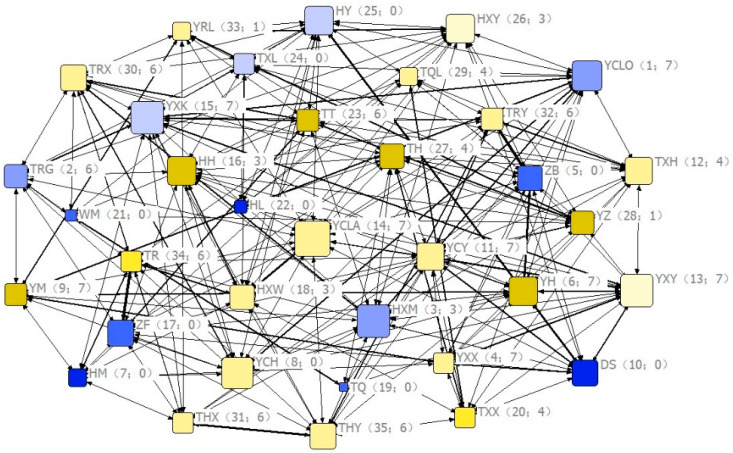
Eigenvector centrality coefficients of group members in the social network. Nodes represent individuals; females and males are shown in yellow and blue tones, respectively; tone gradient (from lowest to highest) indicates age class (1–4); labels represent the name of each individual; node size is proportional to social centrality; the thickness of the links represents its association index in the social network; the numbers inside each pair of brackets indicate social rank and number of relatives, respectively.

Based on the DAI matrix, the social network in different months can be constructed to present the interaction among members of the group intuitively (Figure 3).

We calculated the variables of sex, age class, rank, social centrality, number of relatives, and position of the members from observations. We used individual monkeys as the unit of analysis and collated the average of visual signals for each individual during the monthly collective movement, using five months of repeated measurements in the same focal animals as random effects. Then, a Tobit regression model was used to find that the sex, age, rank, and social centrality had no significant effect on the back-glance signal. Position *(p* = 0.009, coeff ± SE = 0.037 ± 0.015) and the number of relatives (*p* = 0.025, coeff. ± SE = 0.021 ± 0.013) had a significant effect on the back-glance signal (Table 2 and Table 3). On the other hand, sex, age, rank, and the number of relatives had no significant effect on the pause signal, but position (*p* = 0.015, coeff ± SE = 0.035 ± 0.014) and social centrality (*p* = 0.042, coeff ± SE = 0.015 ± 0.008) both had a significant effect on the pause signal (Table 2 and Table 4).

## 4. Discussion

This study explored the distribution, function, and influencing factors of visual signals by analyzing participants’ communication in collective movement. The results showed that about 39% of collective movement used visual signals, and all individuals demonstrated the behavior of back-glance and pause. It was found that back-glance and pause signals from initiators decreased as the number of followers increased. This is in line with prediction 1 (Table 2). It can be confirmed that these two visual signals are indeed the recruitment and monitoring signals. Position, social centrality and number of relatives are the main influencing factors of visual signals.

The back-glance and pause signals emitted by the participating individuals were stronger as the position moves further backward in the troop. This does not support prediction 2 (Table 2), but the contrary is the case. The correlation between visual signals and changes in position has been demonstrated in several species, such as Tonkean macaques [26], Rhesus macaques (*M*. *mulatta*) [26], and white-faced capuchins (*Cebus capucinus*) [39]. However, in all these species, the frequency of visual communication tends to decrease significantly as the following sequences are followed further and further back, in contrast to the results of the present study. We speculate that this phenomenon may be explained by the mating strategies. Individuals at the back of the group tended to be at the edge of the group. When the higher-ranking individuals finish foraging and then leave the feeding site, those lower-ranking individuals secretly look for mating opportunities, deliberately slowing down their subsequent speed, and signaling more frequently to recruit participants from further back. Therefore, maybe the visual signal is not just a recruiting behavior, it is also a desire to mate.

### 4.1. Influence of Sex, Age, and Rank on Visual Communication (Hypothesis 2, Predictions 3–5)

Our study showed that sex did not influence the visual signals in Tibetan macaques. This is not consistent with prediction 3 (Table 2). In some species, pregnant or lactating females, due to their high energy requirements, actively initiate group movements to food, for example, in the white-handed gibbons (*Hylobates lar*) [40], ring-tailed lemurs (*Lemur catta*) [41]. Conversely, in other species, adult males use high-frequency signals to lead groups, typically when there are multiple groups in the same area and the male wants to go to the edge of the group to seek mating opportunities with females from other groups, such as in brown capuchin monkeys (*C. paella*) [42]. Previous studies of Tibetan macaques have found that leadership in this species is not related to sex, possibly because the forest is relatively peaceful and a place that provides shelters for the monkeys’ social activities. After their foraging activities, both males and females were strongly motivated to move from the feeding site to the forest, thus, there is no bias in the frequency of visual signals between genders.

Similarly, visual communication was not affected by age. This does not support prediction 4 (Table 2). In species such as chacma baboons (*Papio ursinus*) and rhesus monkeys, older individuals have more accurate and abundant information about their environment and food resources [43]. They tend to emit signals more frequently to attract the attention of group members, and the group is willing to follow the movements of older individuals because they are aware of their greater experience. However, all the adult macaques in this study had lived in the group for many years, and their daily activity patterns were similar and familiar to their environment. Because of their familiarity with locomotor destinations (e.g., foraging and resting sites), older individuals clearly did not have an informational advantage over younger individuals, which may explain why age did not affect visual signaling in this study.

In the present study, no rank factors were found to influence visual signaling. This does not support prediction 5 (Table 2). In Japanese macaques (*M. fuscata*) [31], highly ranked individuals initiate collective movements more frequently, monitoring individuals behind them by repeatedly looking back in order to compel other members to follow and to increase their own interests. In spider monkeys (*Ateles geoffroyi*) [44], low-rank individuals want to attract other individuals for mating secretly and exhibit more frequent signaling. In contrast, visual signals in Tibetan macaques are shared across all rank levels, suggesting that the species is not absolutely authoritarian, consistent with the findings of distributed leadership in the same study group [19]. We can explain this phenomenon in terms of energy consumption and mating strategy, as too much signaling by high-ranking individuals could cost too much of their own energy, so they would not always emit it. Furthermore, our study was conducted during the mating season (September–January). It also puts a lot of effort into sneaking copulation, with a higher incentive to move from the open fields (i.e., the feeding site) to covert places (i.e., forests).

### 4.2. Influence of Social Centrality on Visual Communication (Hypothesis 2, Predictions 6)

Social centrality significantly affected the frequency of visual signals. The study by Seltmann et al. demonstrated a negative correlation between the strength of social connections and the frequency of back-glance in Barbary macaques [9]. However, in a previous study related to group movements in Tibetan macaques, we found a positive correlation between the strength of social connections and the frequency of successful initiation. Tibetan macaques are a species of " distributed leadership" and have been proposed as an "affiliation-leadership" model [19]. The present study not only further validated this model, but also found that individuals with higher social centrality, who were more connected to group members, were more likely to recruit other individuals through visual signals during collective movements. However, our results showed that social centrality only influenced the pause signal, but not on the back-glance signal. This result only partially supports prediction 6 (Table 2). We speculate that this is because the pause signal usually occurs when foraging is over, during the group departs from the feeding site into the forest and lasts long. Back-glance is not needed to monitor or recruit when a certain number of participants are present [9]. 

### 4.3. Influence of the Number of Relatives on Visual Communication (Hypothesis 2, Predictions 7)

The number of relatives only influenced the frequency of back-glances but not pauses. This result also only partially supports prediction 7 (Table 2), the similar results have been observed in Tonkean macaques and Rhesus macaques [26], namely, the former recruit individuals with affiliation and the latter recruit individuals with kinship. Once a particular individual is recruited, the frequency of the initiator’s back-glance and pause signal decreases significantly [26]. We found that back-glance signals are common in small group movements and mostly in the feeding site with good sightlines and no dense trees. The social context of resting or grooming mostly occurs when related individuals sit together. One of them starts to move, followed by others, and the reaction time of the follower is short [45], without the need for signaling by stopping and waiting.

In this study, two visual communications were recorded due to the limitation of the number of observers. Other communications which can be analyzed in the future work include violently shaking trees, sitting facing a group, walking quickly, and acoustic signals on decision-making. Furthermore, it is necessary to study how pauses and back-glance play their respective roles in collective movement when the number of participants are different.

## 5. Conclusions

We addressed the relationship between visual signals and collective decision-making, and identified what factors affected visual signals. We found that visual signals in Tibetan macaques had the role of recruiting and monitoring members. The more backward the position, the stronger the two visual signals. The higher the social centrality, the stronger the pause signal, and the higher the number of relatives, the stronger the back-glance signal. It gives us a good case study to fit the scale of collective movement. Our study not only confirmed the role of visual communications in collective decision-making, but also investigated the influence of kinship and social relations on individual signals for the first time, providing a reference for the further research on communication behavior in animal societies.

## Figures and Tables

**Table 1:** Ethogram of behaviors recorded during focal animal samples.

Catalog	Definition
Initiator	The first individual that walks more than 10 m in less than 30 s.
Follower	Any individual that walks more than 5 m within 45° in the direction to which the initiator departs before the joining is terminated.
Successful movement	A successful collective movement is recorded when the number of all participants, including the initiator, is equal to or greater than 3.
Termination of joining	When no more individual joins the movement within five minutes after the joining of the last individual.
Back-glance	The individual looks in the direction of other group members, measured as a frequency throughout the movement (i.e., if the individual moves). In the cases, where eyes of animals could not be observed, we used the direction of the head (with an angle wider than 135° with the direction of the movement) to determine a back-glance.
Pause	The individual stops moving for at least 2 s. The frequency of pauses throughout the movement was recorded. The interval of two distinct pauses was more than 2 s.
Proximity	Two or more individuals keep a sitting or lying posture within a certain distance. The distance in this study was 1m.
Aggression	An individual stared, hit on the ground, chased, or bit another individual.
Submission	An individual was attacked by another, but away quickly or fled in opposite direction.

**Table 2:** Summary of analysis outcomes.

Hypothesis	Prediction	Supported by Analysis?
(1) Back-glance and pause are indeed used as communication signals.	(1) The signals will decrease significantly as the number of followers increases.	YES
(2) Discrimination in sex, age class, rank, number of relatives within the group, social network centrality, along with location in the movement queue would influence visual signal emissions.	(2) The further back the position, the weaker the visual signal.	Not supported: the back-glance and pause signals emitted by the participating individuals were stronger as the position moves further back.
	(3) The emission of visual signals differs between females and males.	Not supported: Sex had no effect on visual signals.
	(4) There are variations in the emission of visual signals at different age class.	Not supported: Age class had no effect on visual signals.
	(5) There would be a negative relationship between rank and the frequency of visual signals.	Not supported: Rank had no effect on visual signals.
	(6) Individuals at the core of the social network would emit higher frequency visual signals	Partial supported: Individuals with higher eigenvector centrality coefficient emitted higher frequency of pause signal, but no effect on the back-glance signal.
	(7) A positive relationship would exist between the number of relatives within the group and the frequency of visual signals.	Partial supported: Individuals with more maternal relatives in the group had higher frequency of back-glance signal, but no effect on the pause signal.

**Table 3:** Results of the Tobit regression modelling to test the factors affecting the back-glance signal.

Factor	Coefficient	SE	Z	*p*
Position	0.037	0.015	2.46	<0.01
Sex	−0.290	0.155	−1.87	0.061
Age	−0.038	0.058	−0.65	0.513
Rank	0.009	0.006	1.33	0.183
Centrality	−0.135	0.511	−0.26	0.792
Relatives	0.021	0.013	2.15	<0.05
_cons	0.677	0.261	2.59	<0.01

**Table 4:** Results of the Tobit regression modelling testing the factors affecting the pause signal.

Factor	Coefficient	SE	Z	*p*
Position	0.035	0.014	2.44	<0.05
Sex	−0.169	0.133	−1.27	0.203
Age	0.009	0.049	0.18	0.855
Rank	0.005	0.005	0.88	0.377
Centrality	0.015	0.008	0.59	<0.05
Relatives	0.002	0.022	0.11	0.912
_cons	0.423	0.226	1.87	0.062

## Supplementary Materials

The following are available online at https://www.mdpi.com/2076-2615/11/3/876/s1, Table S1: Attributes of focal animals in YA1 during observation, Table S2: Mean frequency of back-glances and of pauses per collective movement of the first departed individual according to the number of joiners.
