# Supplementary material for: Tibetan Macaques with Higher Social Centrality and More Relatives Emit More Frequent Visual Communication in Collective Decision-Making
Source: Animals (Basel). 2021 Mar 19;11(3):876. doi: 10.3390/ani11030876 (PMC8003505; doi:10.3390/ani11030876)
Supplement: Supplementary file 1 [file animals-11-00876-s001.pdf]

Table S1. Attributes of focal animals in YA1 during observation.

ID	Sex	Age class	Rank	Number of relatives
YCLO	male	young adult group	1	7
TRG	male	young adult group	2	6
HXM	male	young adult group	3	3
YXX	female	young adult group	4	7
ZB	male	middle-aged group	5	0
YH	female	old group	6	7
HM	male	old group	7	0
YCH	female	young adult group	8	0
YM	female	old group	9	7
DS	male	old group	10	0
YCY	female	young adult group	11	7
TXH	female	young adult group	12	4
YXY	female	adolescent group	13	7
YCLA	female	young adult group	14	7
YXK	male	adolescent group	15	7
HH	female	old group	16	3
ZF	male	middle-aged group	17	0
HXW	female	young adult group	18	3
TQ	male	middle-aged group	19	0
TXX	female	middle-aged group	20	4
WM	male	middle-aged group	21	0
HL	male	old group	22	0
TT	female	old group	23	6
TXL	male	adolescent group	24	4
HY	male	adolescent group	25	0
HXY	female	adolescent group	26	3
TH	female	old group	27	4
YZ	female	old group	28	1
TQL	female	young adult group	29	4
TRX	female	young adult group	30	6
THX	female	young adult group	31	6
TRY	female	young adult group	32	6
YRL	female	young adult group	33	1
TR	female	middle-aged group	34	6
THY	female	young adult group	35	6

The rank was calculated using David's Score value; the number of relatives was the number of individuals within a group linked by matrilineal kinship.

Table S2. Mean frequency of back-glances and of pauses per collective movement of the first departed individual according to the number of joiners.

The number of followers	Mean frequency of back-glance	Mean frequency of pause
2	0.33±0.05	0.33±0.07
3	0.43±0.12	0.46±0.1
4	0.52±0.1	0.42±0.08
5	0.5±0.09	0.51±0.06
6	0.5±0.1	0.4±0.04
7	0.37±0.07	0.33±0.07

8	0.27±0.08	0.5±0.09
9	0.29±0.07	0.52±0.04
10	0.29±0.03	0.3±0.04
12	0.17±0.04	0.26±0.1
13	0±0	0.44±0.06
15	0.16±0.08	0.27±0.11
18	0.11±0.05	0.18±0.03
23	0±0	0.1±0.08
